# The role of interventions in secondary hypertension

**Published:** 2016

## Introduction

Interventional therapies can benefit patients with secondary hypertension significantly in respect of mortality and quality of life. Notable causes of secondary hypertension that are amenable to intervention are coarctation of the aorta andrenal artery stenosis (RAS).

Five percent of hypertension derives from secondary causes, Prof Ikechi Okpechi from the University of Cape Town told delegates at AfricaPCR 2016. ‘We need to look for the common clinical clues suggestive of secondary hypertension and investigate accordingly. Work-up requires a clear strategy. A patient history needs to be taken and a physical examination performed. Twenty-four-hour ambulatory blood pressure monitoring is necessary to rule out primary hypertension. Only screen where there is a clinical suspicion and start with simple tests.’

## Coarctation of the aorta

This is an important cause of secondary hypertension and one that is often missed, according to Johannesburg cardiologist, Dr Jeff Harrisberg. ‘The clinical clue is weak or absent femoral pulses. The primary treatments are surgery and/or catheter interventions.’

Indications for coarctation stenting include long-segment coarctation and associated hypoplasia. Covered stents are preferred and balloon angioplasty should be avoided, as it is associated with an uncontrolled response. Possible complications to be aware of include:

dissection, aneurysm and rupture
femoral artery damage
stent migration
cerebrovascular accident or peripheral embolism.

Nice-to-haves when undertaking coarctation stenting include a biplane cath lab, surgical and ICU back up, general anaesthesia, percutaneous femoral artery closure devices and CT angiography. ‘But all of these are not always available in real-world settings. Minimum requirements are a singleplane cath lab with accurate measuring capabilities, the full range of one brand of bare or covered stents, a variety of appropriately sized balloons and large, long sheaths (12–16 Fr)’, concluded Dr Harrisberg.

## RAS

The goal of renal angioplasty and/or stenting for RAS is to provide renal parenchymal protection while controlling blood pressure, which can be challenging, and preventing cardiovascular events. ‘RAS is a relatively uncommon condition, with an incidence of less than 2%’, said Dr Yemi Johnson, from Lagos, Nigeria.

Ultrasound can be a useful investigative tool in good hands, but the healthcare practitioner needs to be trained to look for RAS. ‘Most instances of RAS are unproblematic, so we need clear indications to intervene. These include resistant hypertension, drug intolerance and ischaemic nephropathy.’

Dr Johnson summed up as follows. ‘Renal stenting is a simple procedure if done properly, certainly simpler than coronary artery stenting. It has a high success rate and, while serious complications are possible, these can be prevented with the correct technique.’

